# Possibility for reverse zoonotic transmission of SARS-CoV-2 to free-ranging wildlife: A case study of bats

**DOI:** 10.1371/journal.ppat.1008758

**Published:** 2020-09-03

**Authors:** Kevin J. Olival, Paul M. Cryan, Brian R. Amman, Ralph S. Baric, David S. Blehert, Cara E. Brook, Charles H. Calisher, Kevin T. Castle, Jeremy T. H. Coleman, Peter Daszak, Jonathan H. Epstein, Hume Field, Winifred F. Frick, Amy T. Gilbert, David T. S. Hayman, Hon S. Ip, William B. Karesh, Christine K. Johnson, Rebekah C. Kading, Tigga Kingston, Jeffrey M. Lorch, Ian H. Mendenhall, Alison J. Peel, Kendra L. Phelps, Raina K. Plowright, DeeAnn M. Reeder, Jonathan D. Reichard, Jonathan M. Sleeman, Daniel G. Streicker, Jonathan S. Towner, Lin-Fa Wang

**Affiliations:** 1 EcoHealth Alliance, New York, New York, United States of America; 2 US Geological Survey, Fort Collins Science Center, Ft. Collins, Colorado, United States of America; 3 US Centers for Disease Control and Prevention, Atlanta, Georgia, United States of America; 4 Department of Epidemiology, University of North Carolina, Chapel Hill, North Carolina, United States of America; 5 US Geological Survey, National Wildlife Health Center, Madison, Wisconsin, United States of America; 6 Department of Integrative Biology, University of California Berkeley, Berkeley, California, United States of America; 7 Arthropod-borne and Infectious Diseases Laboratory, Department of Microbiology, Immunology & Pathology, College of Veterinary Medicine & Biomedical Sciences, Colorado State University, Ft. Collins, Colorado, United States of America; 8 Wildlife Veterinary Consulting, Livermore, Colorado, United States of America; 9 US Fish and Wildlife Service, Hadley, Massachusetts, United States of America; 10 School of Veterinary Science, University of Queensland, Gatton, Queensland, Australia; 11 Bat Conservation International, Austin, Texas, United States of America; 12 Department of Ecology & Evolutionary Biology, University of California Santa Cruz, Santa Cruz, California, United States of America; 13 US Department of Agriculture, National Wildlife Research Center, Ft. Collins, Colorado, United States of America; 14 School of Veterinary Science, Massey University, Palmerston North, New Zealand; 15 One Health Institute, School of Veterinary Medicine, University of California Davis, Davis, California, United States of America; 16 Department of Biological Sciences, Texas Tech University, Lubbock, Texas, United States of America; 17 Programme in Emerging Infectious Diseases, Duke-National University of Singapore Medical School, Singapore; 18 Environmental Futures Research Institute, Griffith University, Nathan, Australia; 19 Department of Microbiology & Immunology, Montana State University, Bozeman, Montana, United States of America; 20 Department of Biology, Bucknell University, Lewisburg, Pennsylvania, United States of America; 21 Institute of Biodiversity, Animal Health & Comparative Medicine, University of Glasgow, Scotland, United Kingdom; 22 MRC-University of Glasgow Centre for Virus Research, Glasgow, Scotland, United Kingdom; University of Pittsburgh, UNITED STATES

## Abstract

The COVID-19 pandemic highlights the substantial public health, economic, and societal consequences of virus spillover from a wildlife reservoir. Widespread human transmission of severe acute respiratory syndrome coronavirus 2 (SARS-CoV-2) also presents a new set of challenges when considering viral spillover from people to naïve wildlife and other animal populations. The establishment of new wildlife reservoirs for SARS-CoV-2 would further complicate public health control measures and could lead to wildlife health and conservation impacts. Given the likely bat origin of SARS-CoV-2 and related beta-coronaviruses (β-CoVs), free-ranging bats are a key group of concern for spillover from humans back to wildlife. Here, we review the diversity and natural host range of β-CoVs in bats and examine the risk of humans inadvertently infecting free-ranging bats with SARS-CoV-2. Our review of the global distribution and host range of β-CoV evolutionary lineages suggests that 40+ species of temperate-zone North American bats could be immunologically naïve and susceptible to infection by SARS-CoV-2. We highlight an urgent need to proactively connect the wellbeing of human and wildlife health during the current pandemic and to implement new tools to continue wildlife research while avoiding potentially severe health and conservation impacts of SARS-CoV-2 "spilling back" into free-ranging bat populations.

## Spillover of pandemic viruses

The threat of emerging infectious diseases (EIDs) to wildlife health and biodiversity conservation is recognized [1], but cross-species transmission of novel pathogens, or spillover, is typically viewed in the specific context of originating in a wildlife reservoir and transmitting to humans [2]. Research assessing EID risk has typically focused on identifying geographic regions [3, 4] and wildlife species [5–7] whereby spillover of zoonotic diseases into humans is most likely. Among recent pandemic zoonotic viruses, some have no evidence of transmission back to wildlife or domestic animal populations after establishment in people (e.g., human immunodeficiency virus, which causes acquired immunodeficiency syndrome), while others have repeatedly crossed species boundaries (e.g., pandemic H1N1 influenza A virus) [8, 9]. Evidence of “reverse zoonotic” transmission, sometime referred to as “spillback,” from people to wildlife and domestic animals is widespread [9]; however, systematic surveys to determine the proportion of EIDs that spill back into novel wildlife hosts are lacking. Infection of bats by viruses of probable human origin has been recorded only twice [10, 11], and further transmission [12], or spread to a wider bat population, has not been recorded.

In December 2019, a novel coronavirus was detected from a cluster of 41 atypical pneumonia cases in Wuhan, China, and has since spread to cause a pandemic with significant global morbidity, mortality, and economic impact [13]. Phylogenetic evidence suggests that this virus, severe acute respiratory syndrome coronavirus 2 (SARS-CoV-2), and the clade of SARS-related coronaviruses (SARSr-CoVs) that it belongs in evolved in Old-World bats of the family Rhinolophidae [14–16]. There is no epidemiological evidence of direct or indirect transmission of SARS-CoV-2 from bats to people, but a full genome of its closest known relative (with 96.2% sequence similarity) was reported from an Intermediate Horseshoe Bat (*Rhinolophus affinis*) sampled from Yunnan province, China, in 2013 [17]. The timing of SARS-CoV-2 spillover from bats and any involvement of intermediate host species remain undetermined [18, 19]. The United States currently has the highest number of confirmed human cases of COVID-19, the disease caused by SARS-CoV-2. The consequences of this pandemic are many and include the possibility of SARS-CoV-2 transmission from humans to free-ranging wildlife populations. Given the likely bat origin of SARS-CoV-2, free-ranging bats are a key group of concern for spillover from humans. Humans frequently handle and come into close contact with North American temperate-zone bats during the course of ecological research, wildlife rehabilitation, wildlife/pest control, and disease investigations. Anticipating the need for similar risk assessments across many potentially vulnerable species of wildlife and domesticated mammals globally, we here examine the possibility of humans inadvertently infecting free-ranging North American bats with SARS-CoV-2. We further discuss the possible public health and wildlife conservation consequences of SARS-CoV-2 becoming endemic in bats outside its natural host range.

## Threats of SARS-CoV-2 to North American bats

The pandemic spread of SARS-CoV-2 may directly or indirectly threaten North American bat populations in at least three different ways. First, SARS-CoV-2 might infect any of the diverse and historically isolated 40+ endemic species of temperate-zone North American bats, with or without causing disease, morbidity, and mortality. Second, SARS-CoV-2 might infect and become established in one or more North American bat species, creating novel reservoirs capable of causing human infections (e.g., bat rabies lyssaviruses in the New World [20]). Third, if SARS-CoV-2 infection persists in North American bats of one or more species, it could potentially evolve or recombine with endemic viruses [19, 21] to become more pathogenic or infectious to humans or other animals. In addition to new public health challenges, the latter outcomes could quickly shift public perception of bats from mostly beneficial wildlife with associated disease risks that are manageable to bats posing unacceptable disease risks to human and animal health. Such a shift could increase the likelihood of negative human–bat interactions and conflicts, as well as undermine decades of concerted science, conservation, and education efforts aimed at conserving these valuable animals [22–24]. The potential threat of SARS-CoV-2 transmission from humans to other animals applies to many species of wildlife and domesticated mammals, but the likely bat origin of SARS-CoV-2 and the current threats to bat populations due to another disease in North America influenced us to focus this review on bats.

## Lessons from an epizootic—Susceptibility of North American bats to an introduced pathogen

SARS-CoV-2 is not the first pathogen with the potential for inadvertent spread from people to North American bats. The COVID-19 pandemic follows the arrival of a fungal pathogen (*Pseudogymnoascus destructans*) that as early as 2006 began infecting hibernating bat populations in North America, spreading within and among species to alter the evolutionary trajectory of the continent’s bats [25–28]. Genetic analyses indicate that *P*. *destructans* was introduced to North America [29], in our opinion likely by movement of humans or materials contaminated with fungal spores. White-nose syndrome (WNS), the disease caused by *P*. *destructans*, remains the only documented bat epizootic to cause multiyear, widespread mass mortality [30], although short-term bat die-offs have been also linked to Lloviu virus in Europe [31]. WNS has killed millions of North American bats, affected populations of at least 12 species of 3 genera, and has already spread across half of the US and Canada (whitenosesyndrome.org, accessed 11 May 2020). Effective methods to mitigate WNS spread and impacts remain elusive despite substantial research effort, and targeted mitigation actions have had limited success against its impacts [32]. It took years of concerted international scientific effort to identify the cold-growing fungus, determine that it likely originated somewhere in the temperate zones of Europe or Asia, understand its mechanisms of infection and pathogenicity, develop strategies to limit accidental translocation, and track its rapid spread through an immunologically naïve continental assemblage of hibernating bats [33–35].

The devastating impact of WNS on a diverse group of North American bats likely resulted from evolutionary isolation of the continent’s bat fauna from other parts of the world for millions of years, despite other species of *Pseudogymnoascus* being present. Bats in both Europe and Asia can become infected by *P*. *destructans* but do not suffer mass mortality from WNS [36, 37]. The bat fauna spanning the higher latitudes of North America (in the US and Canada) is composed almost entirely of endemic species belonging to the family Vespertilionidae. Vespertilionid bats occur globally but likely originated and diversified in North America tens of millions of years ago before dispersing to other continents [38, 39]. No extant species of bat in the Americas also occurs outside of the Americas [40, 41], and no bats migrate across the Pacific or Atlantic Oceans [42, 43]. The WNS epizootic demonstrates that a large proportion of these historically isolated bats can be vulnerable to a pathogen introduced from another continent during a single event. Additionally, bats already in a physiologically stressed condition due to WNS or other pressures may be more susceptible to viral infection, experience exacerbated disease outcomes, and/or experience increased viral shedding [44, 45]. The COVID-19 pandemic resembles WNS with respect to potential spread of a pathogen from another continent through interconnected, multispecies assemblages of North American bats that might be immunologically naïve and highlights deficits in our understanding of temperate-zone bat pathogens in North America.

## Gaps in understanding global patterns of Bat–CoV diversity, evolution, and host range

Bats are among the world’s most diverse mammals (comprising approximately 1,400 species [46]), and the global distribution and diversity of CoVs in bats proportionally reflects that of their hosts [47, 48]. Available evidence indicates that bats are natural reservoirs of CoVs, some of which have the potential to cause diseases in humans, domesticated animals, and wildlife [17, 47, 49–59]. Coronaviruses appear to have ancient and ancestral relationships with bats, diversifying globally through a process of within-host evolution and cross-taxonomic host-switching events [47, 59–61]. Bats are the likely mammalian progenitor hosts of all alpha (α-) and beta (β-) CoVs [58, 59, 62, 63] and potentially all coronaviruses [60]. Alpha-CoVs of likely bat origin include the causative agent of swine acute diarrhea syndrome (SADS), which caused mass mortality of over 25,000 piglets on farms in Guangdong province, China [57], and a variant strain of porcine epidemic diarrhea virus (PEDV) that spread rapidly from China in recent decades and caused mass piglet mortality in multiple US states [64]. Human CoVs NL63 and 229E also likely had their evolutionary origins in bats [59, 65]. Two recent human disease epidemics (severe acute respiratory syndrome [SARS] and Middle East respiratory syndrome [MERS]) and now the current COVID-19 pandemic are caused by viruses that probably originated from β-CoVs circulating in bat populations in regions where outbreaks occurred [17, 19, 50–54, 58, 66–68].

The emergence of diseases like SADS, PEDV, SARS, MERS, and now COVID-19 strongly indicates a close association between CoVs that become pathogenic in humans and the wildlife reservoirs from which they originate [17, 50–54, 67]. The evolutionary relationships of CoVs within bats are consistent with geographically structured transmission cycles, with occasional transmission among related bat species [47, 58, 69]. These phylogeographic factors are also universal determinants of viral sharing among all mammals [70]. However, bat–virus association patterns can be particularly difficult to discern because bats often roost together in multispecies aggregations that can facilitate viral sharing, with each species capable of harboring multiple CoV lineages [47, 58, 68, 71]. Host shifts from bats to more divergent taxa are more difficult to predict—firstly, because the potential host breadth for many CoVs is broad [55, 56, 60, 72], and secondly, because host susceptibility and onward transmission involve complex, multistage processes [2, 12]. Bat–CoV associations likely remain substantially undersampled and understudied in temperate-zone North America [47, 71, 73, 74].

## Are viruses like SARS-CoV-2 already present in North American bats?

**Fig 1:**
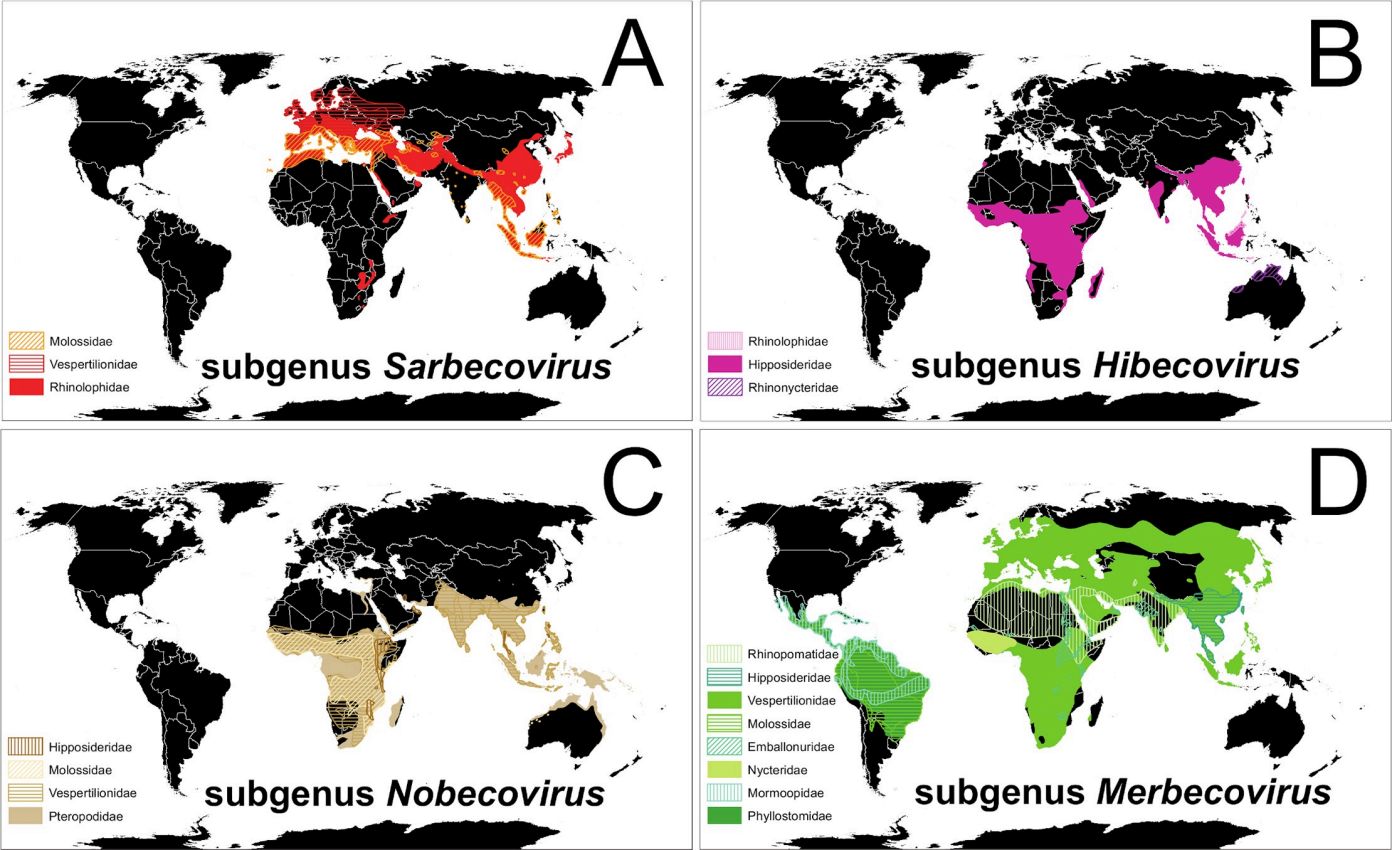
Global patterns of bats and associated β-CoVs. (A) Red-shaded distributions of bat species in which SARS-related β-CoVs of the subgenus *Sarbecovirus* have been detected; (B) pink-shaded distributions of bat species known to host β-CoVs of the subgenus *Hibecovirus*; (C) brown-shaded distributions of bats in which β-CoVs of the *Nobecovirus* lineage have been detected; and (D) green-shaded distributions of bats known to host MERS-related β-CoVs of the subgenus *Merbecovirus*. Different colors and shade styles within each panel represent different families of bats. A data table that includes all known bat species associations for each β-CoVs subgenus and peer-reviewed citations is available at US Geological Survey data release https://doi.org/10.5066/P9U461PJ. *Maps created using ArcMap (ESRI*, *Redlands*, *California*, *United States of America) and bat ranges derived from spatial data on terrestrial mammals from the International Union for the Conservation of Nature (IUCN 2020*. *The IUCN Red List of Threatened Species*. *January 2019 [version 6*.*2]*. *https*:*//www*.*iucnredlist*.*org**; Downloaded on 11 April 2020)*. β-CoV, beta-coronavirus; MERS, Middle East respiratory syndrome; SARS, severe acute respiratory syndrome.

Our examination of CoV evolutionary lineages and global distribution patterns of the diversity of bat species they infect suggests that temperate-zone North American bats could be immunologically naïve to infection by viruses like SARS-CoV-2. Alpha and β-CoVs have been detected in bats on most continents, sometimes with both types occurring in bats of the same species [58, 68]. However, an exception to this pattern is the lack of published evidence that β-CoVs infect bats of temperate-zone North America, despite several search efforts which used methods suitable to detect both α- and β-CoVs [59, 71, 74, 75]. Multiple novel α-CoVs have been detected and described in vespertilionid bats of the US and Canada, infecting species both living in close contact with humans and in remote wild areas [59, 71, 74–76]. However, SARSr-CoVs and β-CoVs of the viral subgenus *Sarbecovirus* have thus far been detected almost exclusively in species of the Old-World Chiropteran suborder Yinpterochiroptera (Fig 1*A*) [47, 58, 69]. The few exceptions to this pattern are the detection of novel Clade 3 and Clade 1 *Sarbecovirus* (*sensu* [53]) viruses in the wrinkle-lipped free-tailed bat (*Mops plicatus*, family Molossidae) in China [77] and the vespertilionid Leisler's noctule (*Nyctalus leisleri*) cohabiting a Bulgarian cave during autumn with several species of rhinolophids in which other SARSr β-CoVs were concurrently detected, suggesting cross-species infections (Fig 1A) [78]. Putative detections of a Clade 1 *Sarbecovirus* were also reported from guano samples of the vespertilionid brown long-eared bat (*Plecotus auritus*) and the molossid European free-tailed bat (*Tadarida teniotis*) on Sardinia, where the same novel β-CoV was described in the greater horseshoe bat (*R*. *ferrumequinum*) [79].

Viruses in the β-CoV subgenera *Hibecovirus* and *Nobecovirus* also have been reported mostly from Old-World bat families Rhinolophidae, Hipposideridae, Rhinonycteridae, and Pteropodidae, except for novel viruses of the latter subgenus detected in four species of the vespertilionid genus *Scotophilus* in Asia and Africa (Fig 1B and 1C) [47, 58, 69].

Bat β-CoVs of the subgenus *Merbecovirus* (MERS-related lineages) occur in a greater diversity of bat families and across more global regions than the other subgenera (Fig 1D) [47, 58, 69]. These widely distributed MERS-like viruses can cause disease in humans (e.g., MERS) and notably appear to be the only bat β-CoVs to diversify among several families of the globally distributed suborder Yangochiroptera (Fig 1*D*) [47, 58, 69].

## Lack of evidence for β-CoVs in temperate-zone North American bats

**Fig 2:**
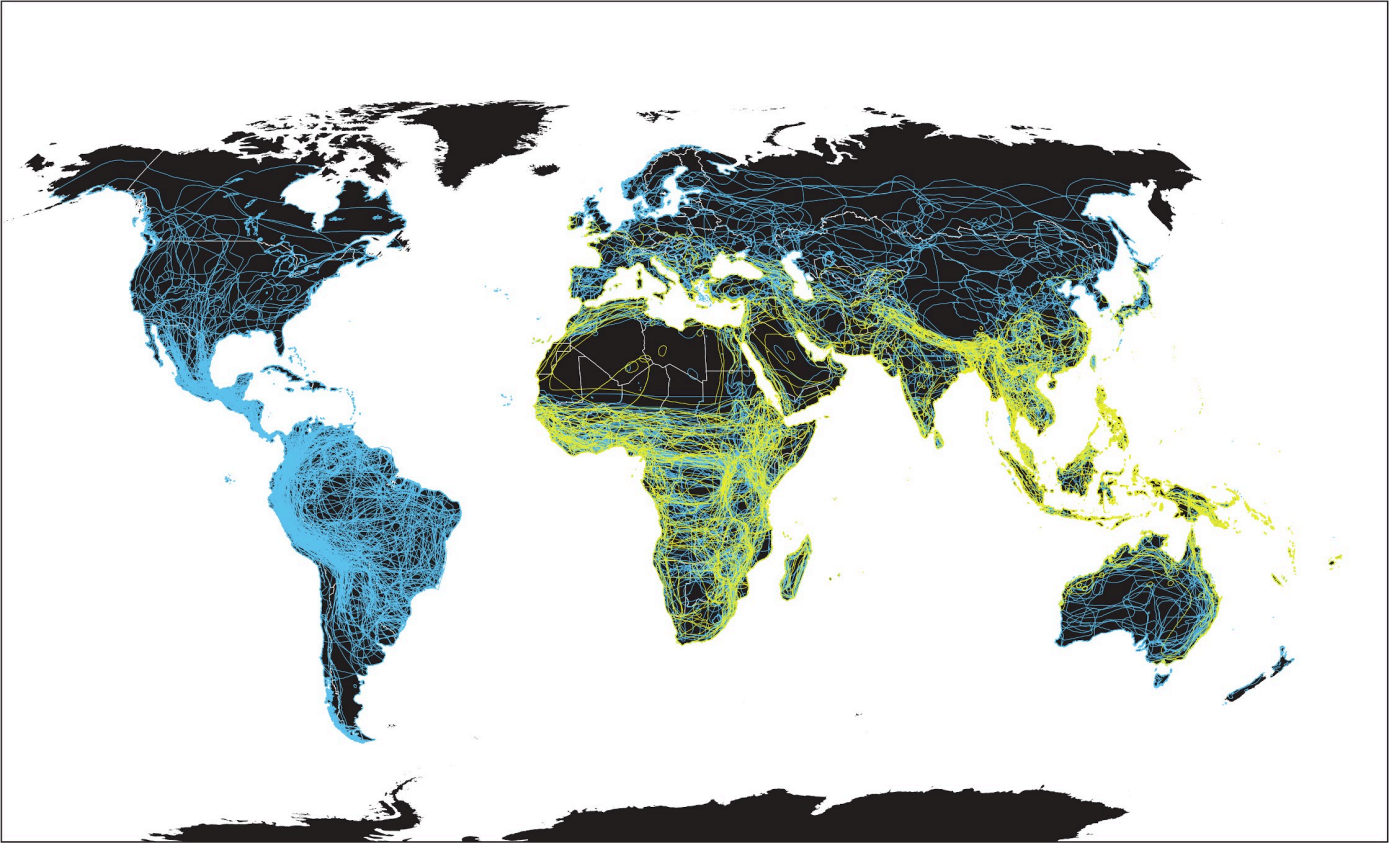
Old-World and New-World bats. Overlapping species distribution outlines of bats in the globally distributed suborder Yangochiroptera (blue) and Old-World Yinpterochiroptera (yellow). *Maps created using ArcMap (ESRI*, *Redlands*, *California*, *USA) and bat ranges derived from spatial data on terrestrial mammals from the International Union for the Conservation of Nature Red List of Threatened Species*, *January 2019 [version 6*.*2]*. *https*:*//www*.*iucnredlist*.*org**; Downloaded on 11 April 2020*.

The several hundred species of extant bats spanning the Americas all belong to the suborder Yangochiroptera, which likely diverged from the Old-World suborder Yinpterochiroptera more than 50 million years ago (Fig 2) [80]. The only β-CoVs detected in the Americas to date belong to the subgenus *Merbecovirus* and appear restricted to two exclusively Neotropical bat families (Phyllostomidae and Mormoopidae) and one that is globally distributed (Molossidae). Distinct CoV lineages in the subgenus *Merbecovirus* were described from three species of *Pteronotus* (family Mormoopidae), four species of *Artibeus*, and Seba’s short-tailed bat (*Carollia perspicillata*; family Phyllostomidae) from tropical regions of Mexico [47, 81]. Novel β-CoVs of the subgenus *Merbecovirus* were detected in two neotropical bat species of the family Molossidae: Wagner’s bonneted bat (*Eumops glaucinus*) in southern Brazil and the broad-eared free-tailed bat (*Nyctinomops laticaudatus*) in southern Mexico [81, 82]. In vitro infections have shown that primary kidney cells from the Jamaican fruit-eating bat (*Artibeus jamaicensis*) can be infected with MERS-CoV, and virus replication and shedding was reported in experimentally infected bats of this species but without obvious clinical signs of disease [83]. Similar to the evidence for natural invasion of bat rabies viruses among New World bats [84], available evidence suggests β-CoVs may have arrived through South America and have long been evolving in Neotropical bats. Although some bat hosts of *Merbecoviruses* overlap geographically with species of temperate-zone North American bats, none occur outside of the Neotropics. Sampling has been limited, but we are not aware of any published detections of *Merbecoviruses* or any other β-CoVs in temperate-zone North American vespertilionid bats.

Our inference of true patterns of CoV occurrence and distribution in bat populations is limited by uneven global sampling. Yet SARSr-CoVs (*Sarbecovirus* spp.), a focus of many surveillance efforts, have been almost exclusively documented in Old-World Yinpterochiroptera. SARSr-CoVs were only found in the ultra-diverse and globally distributed bat suborder Yangochiroptera under conditions with plausible transmission from co-roosting *Rhinolophus* sp. bats [53, 85]. This absence of evidence for SARS-like β-CoVs in yangochiropteran bats in general, and in temperate-zone vespertilionid bats of North America in particular, likely represents a unique biogeographic pattern driven by underlying factors of host susceptibility or life history. These observations also point to the susceptibility of vespertilionid bats under circumstances of SARSr-CoV environmental exposure and that they may not be naturally immune to these viruses.

Bats rank among the most ecologically important mammals and play varied roles in most of Earth’s ecosystems; bats pollinate and disperse seeds of numerous plants in tropical regions, and all over the world, bats are primary nocturnal predators of flying insects [23, 24]. Across the Holarctic, chiropteran species diversity is greatest among hibernating vespertilionid bats. At least 25 of the ecologically diverse vespertilionid species of bats in the US and Canada hibernate [86], which might influence their susceptibility to or interactions with viruses, as has been postulated for common vespertilionids infected with α-CoVs and rabies virus [44, 87–89]. Hibernation strategies vary among species of bats (e.g., degree of sociality, thermoregulatory behaviors, habitat selection) [90], but bat body temperatures during hibernation generally remain consistently below 10º C for periods lasting 7–9 months per year [91], providing a potential mechanism to limit viral replication and spread [92]. Experimental studies to assess the ability of SARS-CoV-2 or other β-CoVs to survive and replicate in bats (cell lines and individuals) at low temperatures [92, 93] would provide additional insight into risk of reverse zoonosis. However, appropriate tools for studying such possibilities are lacking, particularly immortalized cell lines from several hibernating, vespertilionid bats [59]. These tools would also enable interrogation of other physiological features of vespertilionids that may influence susceptibility, such as receptor-binding affinity and the expression of receptors across tissues. Scientists did not discover and isolate the obligately psychrophilic fungus that causes WNS until they collected samples in bat hibernation sites and moved culture dishes for incubation into laboratory refrigerators [25]. Similar innovative explorations outside the typical temperature conditions of laboratory experimentation could help assess the risk of SARS-CoV-2 infecting the more than two dozen species of bats in the US and Canada that hibernate to survive harsh temperate-zone winters.

## Proactively connecting the wellbeing of human and bat populations

Scientists have long recognized the risk of pathogen spillover from humans to bats [94–96], but bat researchers in North America have not systematically addressed this risk prior to WNS. Outside of reservoir host studies, few bat researchers studied infectious diseases in bats before WNS emerged in 2007 [73] nor studied bat viruses (other than rabies) before bats were retrospectively connected to the SARS epidemic [15, 66, 97]. Fortunately, bat and wildlife disease researchers recently began addressing these knowledge gaps in more detail [7, 97, 98]. Possible explanations for why bats might host particularly pathogenic viruses include characteristics of their life history (e.g., long-lived, wide ranging, multispecies aggregations, daily and seasonal heterothermy) [97], unique physiology for repairing their damaged DNA [99], unique ability to suppress some of their innate immunity pathways [100–105], high species diversity [48], and unmatched metabolic range and high body temperatures during flight [106]. Bats also cryptically come into close contact with humans, increasingly in urban and periurban settings as a result of native habitat loss, often crossing human–wildlife interfaces [107–113].

Except for *Lyssavirus* infections, bats rarely show substantial signs of sickness from the same pathogens that cause virulent disease in humans. Bats cope with viral infections in ways that we do not yet fully comprehend, but learning how they do so may reveal important insights to develop therapeutics and ultimately to protect human health [103–105]. In vitro and laboratory studies demonstrate that bats can specifically regulate naïve immunity pathways to effectively cope with viral infection [114]. For example, dendritic cells generated from the bone marrow of the Egyptian rousette (*Rousettus aegyptiacus*) infected with Marburg virus down-regulate immune-stimulatory pathways and maturation of cells targeted by the virus while up-regulating pathogen-sensing pathways [115]. Unique bat immune regulation may occur with MERS-CoV infection, at least under experimental conditions [101]. Egyptian rousette bats experimentally challenged with SARS-CoV-2 by intranasal inoculation became transiently infected, shed virus, and one cohoused bat became infected but showed no clinical signs of disease other than rhinitis [116]. Our potential lack of understanding of clinical signs of illness in bats and the cryptic habits of many species also generally inhibit our ability to easily detect spillover of pathogens from human to bat populations. This may add to uncertainty about cross-species transmission and dispersal of CoVs among human and animal communities. Laboratory findings suggest human viruses that likely originated in bats, such as HCoV-NL63, are capable of infecting bat cells, at least in vitro [59]. SARS-CoV-2 and other CoVs have some of the longest genomes among all RNA viruses, and despite having specialized RNA proofreading machinery [117, 118], they are still prone to recombination and copy errors in hosts, sometimes resulting in functional adaptations (e.g., altered receptor binding capacity or temperature adaptation of enzymes) [119]. CoVs can even recombine with functional fragments of other virus families, such as when a bat-derived CoV gained a functional gene from a reovirus [21]. Spillover of SARS-CoV-2 from infected humans to North American bats they handle or come in close contact with could lead to the virus becoming either less or more pathogenic to bats or other wildlife, domesticated animals, or humans through genetic mixing in one or more novel hosts. The public health and conservation consequences of a more virulent virus could be severe, whereas genetic mixing in a bat host that resulted in a less-virulent virus might go unnoticed.

## Need for an interdisciplinary response

Effectively managing risks of human disease caused by emerging zoonotic pathogens and ensuring the health and conservation of wildlife species that are potential reservoirs of those disease agents can be synergistic goals under a One Health framework. Spillover risk (from or to wildlife) is often greatest in disturbed ecosystems where there is an elevated frequency of human–wildlife interactions or disruption of ecological patterns [3, 120–124]. Thus, effective bat conservation and management requires understanding both pathogens that cause disease in bats, as well as human activities and ecological contexts that increase direct and indirect interactions with bats that could present health risks [2]. Furthermore, fear-based reactions to disease risk from wildlife, such as culling infected bat populations or indiscriminate killing, often have negative unintended consequences for the interconnected health of both humans and bats (e.g., culling of bats in a Uganda mine led to a more than doubling of Marburg virus prevalence in the bats living there) [30, 125–127]. Temperate-zone vespertilionid bats inhabiting human dwellings in the US and Canada represent a particularly relevant human–wildlife interface, in which conservation and management actions to proactively address the potential consequences for pathogen spillover are worth careful consideration [73].

Conservation-compatible surveillance of bat viruses has demonstrated the potential for mutually beneficial collaboration between public health scientists and conservation stakeholders [94, 113, 125, 128, 129]. Disease-focused studies that integrate ecological principles into a rigorous study design provide the most informative context to interpret bat–virus associations and patterns of richness globally [130–132]. Assessing the risks of SARS-CoV-2 spillover into North American bats presents a timely opportunity to form multidisciplinary scientific teams that include experts on emerging infectious diseases and ecologists with expertise on North American bats [128]. Scientists researching emerging infectious diseases can benefit from sampling opportunities and methods that bat researchers have developed for observing, counting, and noninvasively sampling bats [73, 133]. Bat researchers can learn about human and animal health monitoring and supporting laboratory methods, including biosafety, secure handling/transport of CoV-positive samples, and training in the proper use of personal protective equipment (PPE) from professionals with expertise in veterinary and medical sciences [113, 131, 134, 135]. A shared goal of all stakeholders is to identify and implement simple, widely available diagnostic tests for detecting SARS-CoV-2 infection that are species-independent, practical for field and laboratory use, highly specific and sensitive, and that do not require strict biosafety containment [136]. All investigators can also work together to develop mutually beneficial goals, such as joint risk communications to the public with effective and balanced messaging about bat populations and higher risk activities for human–bat contact.

Adopting a precautionary approach in the face of global COVID-19 transmission among human populations, national and international wildlife organizations have advised limiting capturing and handling of bats in the field to minimize the risk of humans infecting wild bats with SARS-CoV-2 until further assessment can be made [137, 138]. The emergence of WNS in 2007 prompted a similar surge in interdisciplinary collaboration that enabled the rapid advances already mentioned and introduced changes to guidance for PPE use and disinfection practices for bat researchers and recreational cavers. Similarly, the emergence of SARS-CoV-2 and other viruses will likely alter the status quo of bat research, emphasizing the need to carefully weigh risks and benefits of wildlife research in the context of population-altering diseases. For example, PPE, including respiratory protection, is a standard practice adopted by many bat virus researchers but by few others studying and regularly handling bats [134, 139]. The urgent research priority of a rapid, quantitative risk assessment and analysis of various mitigation options is currently underway [137, 140]. One key question is whether the proper use of optimal PPE, including bidirectional N95 or equivalent masks, along with effective risk communication and adherence to other basic biosafety practices [134, 141, 142] during field work, can significantly reduce the transmission risk of SARS-CoV-2 from humans to bats. In the interim, until new guidelines are established for handling and for close-proximity work with bats, we have outlined gaps in our understanding of SARS-CoV-2 spillover risks at the interface between humans, domesticated animals, and free-ranging wildlife. Temporarily shifting to “hands-off” bat research methods also seems prudent, wherever possible, and could facilitate ongoing work with reduced risk.

## Examples of “hands-off” research strategies

Multiple research strategies that do not involve close contact with free-ranging bats already exist for addressing critical gaps in understanding CoV diversity, distribution, evolution, and potential health effects in temperate-zone bats. For example, a combination of host-cell receptor analyses and in vitro and in vivo experimental infections across a diversity of bat and other mammalian species have helped inform potential host range expansion for SARS-CoV-2. The receptors that many CoVs use to gain access to host cells, such as angiotensin-converting enzyme 2 (ACE2) and dipeptidyl peptidase-4 (DPP4/CD26), have undergone positive selection in bats, resulting in diverse and recombinant CoV strains [72, 143]. These strains can likely bind to numerous variants of a host receptor protein and facilitate spillover into other animal species [72, 144]. SARS-CoV-2 targets and strongly binds to mammalian ACE2 cell receptors [72, 145, 146]. Beta-CoVs of the subgenus *Merbecovirus* (like those known to occur in the Americas) are not known to target ACE2 cell receptors, instead using as a receptor DPP4/CD26 or possibly other receptors [53, 144]. Current in silico predictions that bats will likely have low susceptibility to SARS-CoV-2 based on ACE2 structural analyses conflict with in vitro evidence and do not comprehensively account for ACE2 amino acid sequence variation (including intraspecific variation) that occurs within bats [17, 72, 145]. Assessing SARS-CoV-2 host range will require additional virus-host receptor binding assays in silico and in vitro [17, 53, 72, 144, 145], together with future experimental infection studies for confirmation of Koch’s postulates. In addition, in vitro studies could evaluate species variability in innate immune responses. These investigations will help quantify the potential for North American bat infection and transmission among free-ranging populations.

Examples of other “hands-off” methods applicable to both bat disease and conservation research include the following: virus discovery and characterization focused on existing specimens archived in scientific museums or through partnerships and collaboration with established national bat disease monitoring or surveillance programs [147, 148]; monitoring echolocation calls to determine the occurrence, distributions, and seasonal or nightly activity patterns of bats [133, 149]; digital imaging methods for counting bats and studying physiology and behaviors in the context of disease [90, 108]; sampling guano from below bat roosts to determine bat species and individual identity, population dynamics, and daily or seasonal patterns of bat occupancy and pathogen shedding [71, 150–152]; and mathematical modeling to predict susceptible host species, virus sharing among hosts, spread patterns, or to estimate mortality in affected populations [5, 70, 122, 135]. Promising areas for innovation include making technologies for bat research more accessible to a broader global user base, less expensive, easier to use, and scientifically reproducible through open-source hardware, software, and laboratory methods [153, 154]. In addition to research, standardized field protocols and probabilistic sampling strategies are needed for monitoring bats and their viruses at continental scales (www.nabatmonitoring.org) [155, 156], as are longitudinal studies across multiple sites to better understand the ecological drivers of CoV dynamics and spillover [157]. Developing simple management tools and methods for rapidly assessing risks of virus spillover from humans to wildlife, while maintaining scientific rigor, could also help with future disease response. It might also be useful to prepare a suite of tools, protocols, and risk communication strategies for natural resource managers and public health officials to immediately deploy while risks are being assessed. Such prepared management resources could include public outreach material and guidelines for enhanced use of PPE for those in closest contact with potentially susceptible wildlife.

## Conclusion

Many questions remain about the risk of SARS-CoV-2 to naïve wildlife populations, the influences of human behavior on those risks, and the potential for establishment of new CoV reservoirs. Cross-species virus transmission events are relatively rare, requiring an infectious reservoir host to be in contact with a recipient host when conditions concurrently favor susceptibility and onward transmission [12, 113, 114]. The currently unknown, but possible and potentially high-consequence, risk of SARS-CoV-2 transmission and establishment in North American bats (or other free-ranging mammals) warrants precaution [116, 140]. Strategically managing interactions between people and potentially susceptible or at risk species can decrease the probability of cross-species virus spillover [113]. Humans that frequently handle and come into close contact with North American temperate-zone bats, such as bat researchers, wildlife rehabilitators, wildlife/pest control workers, and disease investigators, can help decrease any chances of spillover by adopting basic PPE and biosafety practices and carefully evaluating how their actions might adversely affect bat populations. We are at a critical nexus of biosecurity and natural resource conservation that will require ingenuity and diligence to continue important research on bats whilst simultaneously evaluating the ecological future of SARS-CoV-2. Our actions during this current pandemic could profoundly influence and protect the health of both humans and wildlife in North America.

## Supporting information

S1 Table Global patterns of betacoronavirus (β-CoV) associations in bats. The table lists bat species in which betacoronaviruses (β-CoVs) were detected, organized by viral subgenera and clade (for Sarbecorviruses), bat family, bat suborder, and general global region where the species of bat occurs. Reference to the published literature sources of information for each row are listed in the last column. Provided in comma-separated value (.csv) format at https://doi.org/10.5066/P9U461PJ.
(XLSX)
